# The footprint of SARS-COV-2 infection in neonatal late sepsis

**DOI:** 10.1186/s12887-024-04665-7

**Published:** 2024-03-16

**Authors:** Zahra Jamali, Najmeh Mohammadpour, Reza Sinaei, Maedeh Jafari, Fatemeh Sabzevari, Mohammad Hasannejad

**Affiliations:** 1 Department of Pediatrics, School of Medicine, Kerman University of Medical Sciences, Kerman, Iran; 2 Clinical Research Development Unit, Afzalipour Hospital, Kerman University of Medical Sciences, Kerman, Iran; 3 Endocrinology and Metabolism Research Center, Institute of Basic and Clinical Physiology Sciences, Kerman University of Medical Sciences, Kerman, Iran; 4 Department of Pediatrics, Afzalipour Hospital, Kerman University of Medical Sciences, Kerman, Iran; 5 Department of Laboratory Sciences, School of Medicine, Bam University of Medical Sciences, Bam, Iran

**Keywords:** Late sepsis, COVID-19, SARS-COV-2, Neonate

## Abstract

**Background:**

Predicting and finding the viral agents responsible for neonatal late-sepsis has always been challenging.

**Method:**

In this cross-sectional study, which has been done from September 2020 to December 2022, 145 hospitalized neonates suspected to late-onset sepsis alongside routine sepsis workup, were also evaluated for severe acute respiratory syndrome-coronavirus-2 (SARS-COV-2) infection, by nasopharyngeal real-time polymerase chain reaction (RT-PCR) or serological tests.

**Result:**

145 neonates including 81 girls and 64 boys with a mean age of 12.3 ± 5.9 days and an average hospitalization stay of 23.1 ± 15.4 days were enrolled in the study. While 76.6% of them had negative bacterial culture, 63 patients (43.4%) showed evidence of SARS-COV-2 infection in RT-PCR or serology tests. None of the underlying factors including gender, age, and laboratory investigation had a significant relationship with SARS-COV-2 infection. Similarly, the outcomes of death and length of hospitalization were not different between the two groups with positive and negative SARS-COV-2 RT-PCR (*P* < 0.05). There was only a significant relationship between radiological changes including reticulonodular pattern, consolidation, pleural effusion, and different types of infiltrations and SARS-COV2 infection.

**Conclusion:**

Considering the widespread of coronavirus disease 2019 (COVID-19) in newborns, it seems logical to investigate the SARS-COV-2 infection in late-sepsis.

## Introduction

The coronavirus disease 2019 (COVID-19) global pandemic, which was first described in Wuhan, China, has become a global concern in all age groups from pediatrics to geriatrics [1]. Children usually exhibit less severe diseases compared to adults [2]. Although the number of infected neonates seems far less than that of pediatrics and adults [3], neonates are thought to be more susceptible due to their immature immune systems [4]. Both vertical and horizontal transmission modes of SARS-COV2 are relevant in the neonatal period. However, the transmission of the virus to young infants and neonates is thought to occur fundamentally via respiratory droplets during their contact with infected mothers during the post-natal period [5]. Neonatal sepsis that presents in babies under 28 days with several bacterial, viral, and fungal agents is categorized based on infants’ postnatal age at the onset of disease. Most clinicians define late sepsis as occurring at greater than 72 h of life with a range of manifestations demonstrative of infection [6].

Whether more SARS-CoV-2-infected neonates are asymptomatic or whether most of their clinical presentations are non-specific is still an aura of ambiguity. According to the present evidence, the symptoms vary from asymptomatic [5] to the involvement of some organs [7], involvement as multi-systemic inflammatory syndrome (MIS-N) [8], or sepsis-like syndrome (SLS) [5, 9]. Since most of the neonates infected with SARS-COV-2 present with fever, cough, or gastrointestinal symptoms [5], representative of neonatal sepsis, filling the knowledge gaps on the course, severity, therapeutic strategies, and prognosis seems obligatory.

Describing the presentations and biochemical characteristics of SLS in older neonates and infants who tested positive for SARS-CoV-2 infection in comparison to the SARS-CoV-2 negative neonates is of great importance. Although some showed that respiratory symptoms including cough and nasal congestion were more prominent in SARS-COV-2 positive neonates, while poor feeding and lethargy in SARS-COV-2 negative group [5], the differentiation line between COVID-19 and bacterial sepsis remains unclear. Laboratory investigation may not always help distinguish between these two entities. However, in hospitalized neonates blood culture is positive only in 25% of cases and a large number of babies suspected of infection have negative blood cultures and there is no evidence of bacterial pathogen [10, 11]. On the other hand, bacterial co-infection or super-infection that have been previously reported in SARS-COV-2 positive infants [12] may increase the challenges. There is evidence that viral infections like SARS -COV-2 have a significant impact on short-term and long-term complications in hospitalized infants [13–16]. Identifying the diagnosis of COVID-19 in infants and differentiating it from bacterial sepsis can help in the management and spread of coronavirus in this group of infants. Moreover, most of the available information has been obtained from previous studies that are related to the infections caused by previous SARS infections. There is no accurate and complete information about COVID-19 infection in infants. Here, we investigate the SARS-COV-2 infection in the context of late sepsis.

## Method and materials

### Study design and data source

This descriptive cross-sectional study was conducted at a COVID-19 dedicated tertiary care referral hospital from September 2020 to December 2022, during the COVID-19 pandemic. We carried on a single-center study in the neonatal intensive care unit (NICU) of the Afzalipour hospital in Kerman, southeast of Iran. This study has been approved by the local ethics committee. Parents were requested for informed consent on admission.

The inclusion criteria were term and late preterm neonates who presented with late onset sepsis. Exclusion criteria included all neonates under three days and above 28 days of age.

145 infants suspected of late-onset sepsis who were more than three days old, either following birth or later in life, were included in the study. Babies who were suspected of late onset sepsis had several symptoms, such as lethargy, poor feeding, fever, respiratory distress and failure, vomiting, etc. The infants’ clinical and demographic characteristics, laboratory markers, and clinical presentations were immediately recorded.

Intravenous antibiotics was initiated based on the clinician’s judgment. Blood, cerebral spinal fluid (CSF) and urine cultures sampling was compulsory prior to starting antibiotics.

Enrolled infants were tested for the SARS-COV-2 RT–PCR on nasopharyngeal swab and collected samples were analyzed for detecting virus at local Institute for Virology. Viral RNA was extracted using an automated nucleic acid isolation system (Zybio, EXM6000) according to manufacture manual and the product was processed after that. Detection of SARS-CoV-2 by one stage real time-PCR (RT-PCR) was performed, using the current kits used in Iran (http://pishtazteb.com/en/products/molecular-kits/covid-19-one-step-rt-pcr) according to the manufacture’s protocol. At the same time, serology for Immunoglobulin-M (IgM) SARS-COV-2 was sent as well. Similarly, detection of SARS-CoV-2 antibodies was performed using SARS-CoV-2 immunoglobulin M (IgM) ELISA kits (Pishtaz Teb, Iran, http://pishtazteb.com) and SARS-CoV-2 IgG ELISA kits (Pishtaz Teb, Iran http://pishtazteb.com) according to the manufacturer’s protocol (Specificity: 97.30%; Sensitivity: 79.40%) [17]. Clinicians were not blinded to the results of the SARS-COV-2 tests and in case of a SARS-COV-2 positive test, infants were immediately isolated in the special COVID-19 unit.

### Statistical analysis

For qualitative variables, frequency and percentage were used. For quantitative variables, if the distribution was normal, the mean and standard deviation (SD) were used; and if it was not normal, the median and interquartile range were used. Comparing the frequency of the virus in the two studied groups, the chi-square test was performed. In order to compare the serum levels of the studied antibodies in both groups; if the distribution of the data was normal, the student-T test was used and if was not normal, non-parametric tests were used. For evaluation of the normality of the data, the Kolmogorov-Smirnov test and Shapiro Wilk test was performed.

Data were analyzed using IBM SPSS Statistics for Windows, Version 21.0. Armonk, NY: IBM Corp. A two-sided *P*-value less that 0.05 was considered significant.

## Results

Among 145 participants, the median age was 10 days (IQR 8–14), including 81 girls (55.9%) and 64 boys (44.1%). Median gestational age was 32 weeks (IQR 28–37), as described in method section, enrolled in the study. The median length of hospital stay was 19 days (IQR 14–30).

While about three quarters (76.6%) of fluid samples were culture negative, 21 (14.5%), 15 (10.3%), and 2 (4.1%) had positive cultures in blood, urine, and CSF specimens, respectively.

41 out of 145 patients (28.3%) had only positive SARS-COV-2 RT-PCR tests, and 12 patients (8.3%) had positive SARS-CoV-2 Immunoglobulin-M (IgM) results, and 10 patients (6.9%) were positive in both tests.

Only 6.2% of cases did not need respiratory care, 70 infants (48.3%) intubated and required invasive ventilator support, 47 patients (32.4%) were supported non-invasively with continuous positive airway pressure (CPAP) during their admission, and 19 patients (13.1%) were received oxygen by head box.

The median C-reactive protein (CRP) value was 10 mg/dL (IQR 1.9–21).

Regarding other laboratory abnormalities including: Hyponatremia (< 135 mEq/L), Hyperkalemia (> 6 mEq/L), Hypocalcemia (< 8.5 mg/dL), Leukocytosis (> 11.0 × 10^9^/L), Leukopenia (< 4 × 10^9^/L), Thrombocytosis (> 450 × 10^9^/L) and Thrombocytopenia (< 150 × 10^9^/L), which was shown in Table 1, no significant difference was seen in both groups.

**Table 1:** The relation between SARS-CoV-2 RT-PCR result with laboratory and radiological findings

Variable		COVID-19 PCR Results				*P*-Value
		Positive		Negative		
		Frequency	percent	Frequency	Percent	
Sodium	Hyponatremia	5	17.2	24	82.8	0.14
	Normal (135–145 mEq/L)	36	31	80	69	
Potassium	Hyperkalemia	1	11.1	8	88.9	0.44
	Normal (3.9–5.9 mEq/L)	40	29.4	96	70.6	
Calcium	Hypocalcemia	8	27.6	21	71.4	0.92
	Normal (8.5 to 10.2 mg/dL)	33	28.4	83	71.6	
WBC	Leukocytosis	6	40	9	60	0.37
	Leucopenia	2	15.4	11	84.6	
	Normal (4.5 to 11.0 × 10^9^/L)	33	28.2	84	71.8	
hematocrit	Low	1	6.7	14	93.3	0.067
	Normal	40	30.8	90	69.2	
Platelet	Thrombocytosis	4	20	16	80	0.65
	Thrombocytopenia	4	26.7	11	73.3	
	Normal (150–450 10^9^/L)	33	30	77	70	
Radiological changed	Reticulonodular	27	65.9	33	31.7	0.001<
	Consolidation	2	4.9	13	12.5	
	Pleural effusion	0	0	11	10.6	
	Diffuse infiltration	0	0	3	9.2	
	Patchy infiltration	11	28.6	21	20.2	
	Normal	1	2.4	23	22.1	
**Variable**		**median**	**IQR**	**median**	**IQR**	*P*-Value
CRP (mg/dl)		10	1.45 to 16	10	2.35 to 21	0.75

Only 24 patients (16.6%) had normal chest radiographs, the others were experienced a wide spectrum of findings including reticulonodular pattern, consolidation, diffuse infiltration, patchy infiltration, and pleural effusion that were found in 60 (41.4%), 15 (10.3%), 3 (2.1%), 32 (22.1%), and 11 (7.6%), respectively.

31 patients (21.4%) had a history of close contact with COVID-19 patients. In addition, 77 patients (53.1%) had some comorbidities. (History of congenital heart disease, Respiratory Distress Syndrome, and gastrointestinal problems, such as malrotation, Hirschsprung’s disease, Necrotizing Enterocolitis, etc.)

Comparing the RT-PCR positive in all fluid cultures subgroups, the relationship between all cultures, especially urine cultures and the molecular investigation of SARS-COV-2 was significant (Table 2).

**Table 2:** Comparison between SARS-CoV-2 RT-PCR results and body fluid cultures

Variable		COVID-19 PCR Results				*P*-value
		Positive		Negative		
		Frequency	percent	Frequency	Percent	
Blood culture	Positive	4	19	17	81	0.31
	Negative	37	29.8	87	70.2	
Urine culture	Positive	0	0	15	100	0.01
	Negative	41	31.5	89	68.5	
CSF culture	Positive	0	0	2	100	0.37
	Negative	41	28.7	102	71.3	
All Cultures	At least one + culture	4	11.8	30	88.2	0.015
	All of culture _	37	33.3	74	66.7	

Association between SARS-CoV-2 infection (RT-PCR results) with underlying factors is presented in Table 3, which none of the gender, neonatal age, gestational age, and close contact were related to molecular results (*P* > 0.05).

**Table 3:** Comparative clinical and demographic data between SARS-CoV-2 RT-PCR results with underlying factors in enrolled infants

Variable	COVID-19 PCR Results				*P*-value
	Positive		Negative		
	Frequency	percent	Frequency	percent	
Gender					0.48
Female	21	51.2	60	53.7	
Male	20	48.8	44	42.3	
Close contact					0.31
Yes	11	35.5	20	64.5	
No	30	26.3	84	73.7	
**Variable**	**median**	**IQR**	**median**	**IQR**	***P*** **-value**
Gestational age (week + day)	35	29 + 5 to 37	31	28 to 36	0.21
Newborn age (day)	10	8 to 14	10	8 to 14	0.21

Comparing between SARS-CoV-2 infections (RT-PCR result), laboratory parameters alterations, and radiological findings are summarized in Table 1. There is only a significant relationship between radiological changes and COVID-19 (*P* < 0.001). There is no significant correlation between other laboratory and clinical alterations and SARS-CoV-2 infection.

Similarly, other parameters including the length of hospital stay, comorbidities, ventilator support, and the outcome of death had no relationship with RT-PCR positivity (Table 4).

**Table 4:** The relation between SARS-CoV-2 RT-PCR result and course, comorbidities and outcome

Variable		COVID-19 PCR Results				*P*-Value
		Positive		Negative		
		Frequency	percent	Frequency	Percent	
Respiratory support	Intubation	21	30	49	70	0.26
	CPAP	15	31.9	32	68.1	
	Head box	5	26.3	14	73.7	
	No respiratory support	0	0	9	100	
Outcome	Expire	5	23.8	16	76.2	0.59
	Recovery	36	29.5	86	70.5	
Comorbidities	Yes	22	28.6	55	71.4	0.97
	No	19	28.4	48	71.6	
**Variable**		**average**	**Standard deviation**	**Average**	**Standard deviation**	*P* **-Value**
Duration of hospitalization		22.8	12.41	23.44	16.5	0.39

## Discussion

In this study, more than three quarters of all fluid samples of patients suspected to late-onset sepsis were culture negative, while 63 patients (43.4%) showed molecular or serological evidence of COVID-19. There was a significant correlation between all cultures and urine cultures in two groups with positive and negative RT-PCR tests. There was a significant relationship between radiological changes and COVID-19. The outcomes of death and hospitalization stay were different between the two groups.

Neonatal late onset sepsis is one of the main causes of mortality in intensive care units (ICU), characterized by multiple organ dysfunction due to an uncontrolled response to infection. Various pathogens, including bacteria, fungi, viruses, and even parasites cause sepsis by triggering aberrant immune responses in multiple organs, especially the lungs [18]. Therefore, diagnosis of SARS-CoV-2 infection in the NICU is an important issue that helps in successful antibiotic management, provided that certain guidelines can be prepared, although evidence is still lacking about the exact virus incidence. The passible benefits of virus detection may come from early implementation of effective isolation and infection control to avoid virus spread.

A positive microbiological fluid culture poses the gold standard for the diagnosis of neonatal sepsis. However, a high number of culture-negative cases in blood samples, and the use of other fluid cultures, especially urine cultures remains controversial [18, 19].

There are studies regarding the frequency of SARS-COV-2 infection among the infants with sepsis. However, the number of these studies is limited, especially in neonates with late-onset sepsis. Making the definite diagnosis of viral sepsis is particularly challenging. In a study of infants aged between 1 and 90 days with sepsis, bacterial agents were found in only nearly 15% of cases, making viral infection more likely as a plausible cause of sepsis in these patients [20]. Young infants have significantly reduced Tall- like receptor expression, antigen-presenting call activity, Natural Killer cell responsiveness, T-cell functionality, complement concentration and B-cell maturity, raising the risk of severe disease from viruses in this age group [21]. Therefore, viral sepsis would be more accurate to describe the clinical manifestations of severe cases of COVID-19 [13].

Several studies reported diagnosis and management of COVID-19 sepsis in the early stage of the neonatal period [14–16, 22], but less paid surrounding COVID-19 in neonates suspected to late-sepsis mainly as sporadic reports [5, 6, 23–31]. Certainly, bacterial co-infectious should not be forgotten [32]. A negative bacterial culture with positive COVID 19 result sounds like a good opportunity to discontinue or deescalate the antimicrobials, where some was performed a partial septic work up in their patients [5].

Although, the potentially acquired virus is usually from close contacts, especially with caregivers [18], only 31 out of our 145 neonates (21.4%) had a history of definite close contact with confirmed COVID-19 patients. However, only 11 out of 31 patients infected with SARS-C0V-2 infection. The relation between COVID-19 and close contact history was not significant. The incidence of transmission through house had contact among children has been estimated between 45% and 91% [29]. Similarly, in an Indian study, no identified contact source was found in half of SARS-COV-2 infected neonates [33].

Although chest X Ray imaging plays an important role in the evaluation of neonatal late sepsis, there is limited information regarding COVID-19 patients. In present study, there was significant relationship between radiological changes and COVID-19. On their review, Mirnia et al. found that mild pulmonary infection and ground glass appearance were the most common chest radiographs findings [34]. Also Limavady et al. suggested that COVID-19 severity are statistically significantly associated with an increased risk of abnormal CXR findings [35].

It is suggested that if the infant has a negative bacterial culture but positive radiological changes, COVID-19 tests can be repeated for these specific babies to make a final diagnosis during COVID-19 pandemic.

There was no significant correlation between laboratory and clinical alterations and SARS-CoV-2 infection. Zhu et al. discussed that the SARS-CoV-2 in neonates can develop to respiratory distress and death. Thrombocytopenia and liver function abnormalities were among affected laboratory parameters in their study [36]. Lymphopenia, hypocalcemia, hypokalemia, thrombocytopenia, elevated LDH and INR were reported in Kaveh and colleagues study. Elevated inflammatory markers were not common in their study [15]. Similarly, fewer inflammatory marker abnormalities including CRP were found in Saeedi and colleagues’ study [34]. Some studies showed that chest radiographs in neonates could be normal and may show lung consolidation, ground glass opacities, and patchy infiltrates especially in sub-pleural areas [16, 34].

## Conclusion

In conclusion, none of the underlying factors (gender, age, and laboratory parameters); the outcomes of death and length of hospitalization were not different between the two groups with positive and negative SARS-COV-2 RT-PCR. There was only a significant relationship between radiological changes including reticulonodular pattern, consolidation, pleural effusion, and different types of infiltrations and SARS-COV2 infection.

Although there is a considerable amount of evidence available regarding the role of viral agents in neonatal sepsis, especially during the COVID-19 pandemic, there are still many questions regarding the causative role of SARS-CoV02 infection in neonatal sepsis. Regarding the role of - SARS-CoV-2 in mimicking or exacerbating the symptoms of late sepsis in infants, additional implementation of SARS-CoV-2 RT-PCR assays into clinical practice may provide a significantly improved tool to diagnose mentioned viral infection. The mentioned radiological findings are of great importance.

## Data Availability

The datasets used and/or analyzed during the current study are available from the corresponding author on reasonable request.
